# Effectiveness and cost-effectiveness of treatment with additional enrollment to a homeopathic integrated care contract in Germany

**DOI:** 10.1186/s12913-020-05706-4

**Published:** 2020-09-15

**Authors:** Benjamin Kass, Katja Icke, Claudia M. Witt, Thomas Reinhold

**Affiliations:** Institute for Social Medicine, Epidemiology and Health Economics, Charité - Universitätsmedizin Berlin, corporate member of Freie Universität Berlin,Humboldt-Universität zu Berlin, and Berlin Institute of Health, Berlin, Germany

**Keywords:** Cost analysis, Chronic disease, Cost-effectiveness analysis, Germany, Health care, Costs; Homeopathy; Retrospective Studies.

## Abstract

**Background:**

A number of German statutory health insurance companies are offering integrated care contracts for homeopathy (ICCHs) that cover the reimbursement of homeopathic treatment. The effectiveness and cost-effectiveness of these contracts are highly debated.

**Methods:**

To evaluate the effectiveness and cost-effectiveness of treatment after an additional enrollment in an ICCH, a comparative, prospective, observational study was conducted in which participants in the ICCH (HOM group) were compared with matched (on diagnosis, sex and age) insured individuals (CON group) who received usual care alone.

Those insured with either migraine or headache, allergic rhinitis, asthma, atopic dermatitis and depression were included. Primary effectiveness outcomes were the baseline adjusted scores of diagnosis-specific questionnaires (e.g. RQLQ, AQLQ, DLQI, BDI-II) after 6 months. Primary cost-effectiveness endpoints were the baseline adjusted total costs from an insurer perspective in relation to the achieved quality-adjusted life years (QALYs). Costs were derived from health claims data and QALYs were calculated based on SF-12 data.

**Results:**

Data from 2524 participants (1543 HOM group) were analyzed. The primary effectiveness outcomes after six months were statistically significant in favor of the HOM group for migraine or headache (Δ = difference between groups, days with headache: − 0.9, *p* = 0.042), asthma (Δ-AQLQ(S): + 0.4, *p* = 0.014), atopic dermatitis (Δ-DLQI: − 5.6, *p* ≤ 0.001) and depression (Δ-BDI-II: − 5.6, p ≤ 0.001). BDI-II differences reached the minimal clinically important difference. For all diagnoses, the adjusted mean total costs over 12 months were higher in the HOM group from an insurer perspective, with migraine or headache, atopic dermatitis and depression suggesting cost-effectiveness in terms of additional costs per QALY gained.

**Conclusion:**

After an additional enrollment in the ICCH, the treatment of participants with depression showed minimally clinically relevant improvements. From an insurer perspective, treatment with an ICCH enrollment resulted in higher costs over all diagnoses but seemed to be cost-effective for migraine or headache, atopic dermatitis and depression according to international used threshold values. Based on the study design and further limitations, our findings should be considered cautiously and no conclusions regarding the effectiveness of specific treatment components can be made. Further research is needed to overcome limitations of this study and to confirm our findings.

**Trial registration:**

clinicaltrials.gov, NCT01854580. Registered 15 March 2013 – Retrospectively registered, https://clinicaltrials.gov/ct2/show/NCT01854580

## Background

The (cost-) effectiveness of homeopathic treatment and its reimbursement by health insurance companies are part of ongoing debates [1–4]. The main overall concern is the high dilution of original substances to a level at which no molecule of these substances can be found and the lack of valid scientific explanations of its mechanisms of action [1, 2]. Despite this debate, many patients seek additional complementary medicine treatments for their disease, for example, homeopathic treatment [5, 6]. Moreover, to date, approximately two-thirds of German statutory health insurance companies are offering and at least partially reimbursing their insured clients for a variety of different additional homeopathic treatment opportunities [7]. For this purpose, German statutory health insurance companies developed homeopathic integrated care contracts with homeopathic physicians and their representing management company [7]. In two previous cost comparison studies of our research group using routine data from a large German statutory health insurance company (Techniker Krankenkasse), it was shown that treatment after an additional enrollment in an integrated care contract for homeopathy (ICCH) comes with long-term cost increases from both the insurer and societal perspectives [8, 9]. However, both studies only analyzed cost data. Conclusions about the effectiveness and cost-effectiveness were impossible to draw from these analyses since the effectiveness was not measured. The latest systematic review, which included 14 studies regarding the cost-effectiveness of homeopathic treatment, showed methodological weaknesses in combination with heterogeneous studies and resulted in the inability of the authors to reveal “any firm conclusions about the cost-effectiveness of homeopathy based on the existing evidence” [10]. Another problem was the relatively small sample sizes of the included studies, which has occurred in previous studies as well [11, 12].

Therefore, the aim of the present study was to compare the effectiveness and cost-effectiveness of the treatment after an additional enrollment in the ICCH to a matched control condition receiving usual care alone for selected chronic diseases.

## Methods

### Study design and intervention

A two-arm comparative, prospective, observational study was designed to evaluate the effectiveness and cost-effectiveness after an additional enrollment in the ICCH of the German statutory health insurance company in comparison to insured individuals who did not participate in the ICCH for a period of 12 months. Participants of both groups were free to receive usual care, ICCH related treatment in the intervention group was additionally offered. The ICCH includes an initial consultation with a detailed homeopathic case history and case analysis, including repertorisation and advice. It also includes follow-up consultations. Homeopathy within this contract must be performed by certified physicians. A more detailed description of the ICCH was given in our previous publication by Ostermann et al. 2017 [9]. After obtaining the informed consent, study participants could be included if they were diagnosed either with allergic rhinitis (ICD-10 J30), asthma (J45), atopic dermatitis (L20), depression (F32), headache or migraine (R51 & G43). Study diagnoses were chosen based on the expected sample sizes with the aim to display the variety of chronic diseases typically treated also by homeopathic physicians. Participants’ enrollment started in March 2013, and the last follow-up questionnaires were received in January 2017. The economic data were available for analyses in July 2018. The study was officially registered (ClinicalTrials.gov registry: NCT01854580) and was approved by the Ethics Committee of the Charité - Universitätsmedizin Berlin (EA2/121/12).

### Study population

Participants in the intervention group (HOM group) were recruited in the medical practices of participating homeopathic physicians after newly subscribing to the ICCH. The matched controls (CON group) were directly contacted by the insurance company. For each participant in the HOM group, up to 20 potential CON participants were contacted who had the same diagnoses (recorded from self-declaration via the screening website and validated with retrieved ICD-10 codes in the health claims data), age (in years) and sex; these individuals were invited to participate as controls in the study.

In addition to being diagnosed with and under treatment for one of the study diagnoses, all participants needed to speak German, have regular access to the Internet (for receiving e-mails during the study) and be insured for at least 12 months prior to baseline. Participants diagnosed with either allergic rhinitis, asthma or atopic dermatitis needed to be ≥12 years of age. When diagnosed with either depression, migraine or headache they needed to be ≥17 years of age. Potential participants of both groups were excluded from the analysis if they (a) were diagnosed with any type of cancer requiring treatment, (b) were enrolled in a disease management program, (c) were enrolled in another study at baseline or (d) if their self-stated study diagnosis was not confirmed within their health claims data later. Inclusion and exclusion criteria were checked using a web-based screening questionnaire prior to baseline.

### Effectiveness and cost-effectiveness outcomes

Independent analyses for each diagnosis were performed. The effectiveness was assessed using disease-specific measurement instruments at baseline and at 3, 6 and 12 months in both groups [13–22]. Baseline was defined as the individual date of signing the informed consent. Table 1 gives an overview of the measurement instruments used. The primary effectiveness endpoints were the baseline adjusted scores of the diagnosis-specific effectiveness measures 6 months after study onset to evaluate whether earlier clinical improvements after 6 months occur. Within the HOM group, baseline questionnaires should have been completed before the initial homeopathic anamnesis. A priori, we further planned specific analyses for adolescent participants with allergic rhinitis, asthma and atopic dermatitis. All questionnaires were web-based, and participants received personalized links.

**Table 1:** Description of applied diagnosis-specific measurement instruments

Diagnosis (*ICD*-*10*-Code)	Measurement instruments
Migraine/headache *(G43/ R51)*	
	Days with headache (last 4 weeks) [13]
	*Scale range from 0 to 28 days*
Allergic rhinitis *(J30)*	
	Standardised Rhinoconjunctivitis Quality of Life Questionnaire (RQLQ(S)) [14] [for participants aged ≥18 years]
	Adolescent Rhinoconjunctivitis Quality of Life Questionnaire (AdolRQLQ) [17] [for participants aged 12–17 years]
	*Scale range from not impaired at all [0 points] to severely impaired [6 points]* *Minimal clinical important difference for both questionnaires: ±0.5 points*
Asthma *(J45)*	
	Standardised Asthma Quality of Life Questionnaire (AQLQ(S)) [15] [for participants aged ≥17 years]
	Paediatric Asthma Quality of Life Questionnaire *– Standardised version* (PAQLQ) [16] [for participants aged 12–16 years]
	*Scale range from not impaired at all [1 point] to severely impaired [7 points]* *Minimal clinical important difference for both questionnaires: ±0.5 points*
Atopic dermatitis *(L20)*	
	Dermatology Life Quality Index (DLQI) [18] [for participants aged ≥17 years]
	Children Dermatology Life Quality Index (CDLQI) [19] [for participants aged 12–16 years]
	*Scale range from no effect on patient’s life [0 points] to extremely large effect on patient’s life [30 points]* *Minimal clinical important difference: ±4 points*
Depression *(F32)*	
	Beck Depression Inventory II (BDI-II) [20, 21]
	*Scale range from minimal depression symptoms [0 points] to severe depression symptoms* *[63 points]* *Minimal clinical important difference: ±5 points*
All participants	
	12-Item Short Form Health Survey Version 1 (SF-12) [22]
	Online Screening questionnaire (for checking inclusion end exclusion criteria)

The primary endpoints of the cost-effectiveness analysis were the total costs from an insurer perspective in relation to the achieved quality-adjusted life years (QALYs) for the 12-month observation period after baseline. If statistically significant higher QALYs and total costs in the HOM group were found, the incremental cost-effectiveness ratio (ICER) was calculated [23]. Data on medical resource consumption and incurred costs were calculated on an annual level and extracted from pseudonymized routine health claims data provided by insurance company for each participant for the year prior to and after baseline. Total costs included costs of the ICCH (only the HOM group), medication costs, outpatient care costs, inpatient costs and other costs (i.e., costs for home health care, patient transportation, remedies and aids). Due to the one-year observation period, costs and effects were not discounted. Full-year QALYs were calculated based on the data of the Short Form Health Survey (SF-12) at baseline and at 3-, 6- and 12-month follow-ups. Thus, an algorithm developed by Brazier and Roberts [24] was applied to convert the SF-12 data into SF-6D health state utility values. Assuming linear changes between the longitudinal utility values, the area under the curve was used to calculate QALYs [25]. The calculation of QALYs requires existing SF-12 data at baseline and for at least one further follow-up. If these requirements were not met, QALYs were not calculated, and these participants were not considered in the cost-effectiveness analysis. Furthermore, for the extrapolation of missing utility values, unchanged, constant utility values were assumed if no further utility value was available after the missing value (last observation carried forward).

For secondary analysis, the diagnosis-specific cost-effectiveness after 12 months was considered from a societal perspective. Hence, productivity losses due to absence from work as well as from health claims data derived co-payments were added to total costs. The human capital approach and a daily gross mean value of 239.20€ (basis 2014) were used as described in our previous cost comparison analyses to calculate productivity losses [8, 26, 27]. The enrollment to the ICCH and treatment within the contract were free of charge for the participants. Further potential out-of-pocket costs were not inquired.

### Statistical analysis

Baseline data were reported descriptively as proportions or means and standard deviations (SDs). For all outcome analyses, adjusted means (and 95% confidence intervals (CIs)) and the mean differences between the HOM and CON groups were determined using separate covariance analysis (ANCOVAs) adjusted for age, sex, school education, state of residence, use of complementary medicine in the 12 months prior to baseline (binary variable, self-reported), baseline utility value and protocol-compliant initial anamnesis. The latter was included as a covariate because approximately two-thirds of the participants in the HOM group (67.1%) received their initial homeopathic anamnesis in contrast to the study protocol before completing the baseline questionnaires. Cost outcomes were further adjusted for the total costs for the 12-month period prior to baseline. The diagnosis-specific effectiveness outcomes were adjusted for the baseline values of the respective measurement instrument. All analyses were performed in a diagnosis-specific manner, based on an Intention-to-treat approach. Group membership was determined only for the enrollment in the ICCH, independent of receiving homeopathic treatment afterwards.

A priori it was assumed that small to moderate group differences corresponded to a standardized mean difference of 0.3. This decision was based on clinicians’ expert opinions and reflects a standardized effect size that is a lower limit of moderate clinical relevance. At a determined significance level of 0.05 (two-sided test), 176 participants per group were required per diagnosis for a power of 80%. With drop-outs, an inclusion of 220 participants per group and diagnosis was planned. Initially, pairwise matched comparisons were planned, but due to recruitment problems in both groups, the analyses were changed to group mean comparisons.

All tests performed were double-sided with a significance level of 0.05. No specific strategies for outlier handling were used, since the underlying cost data showed no hint for extreme outliers. Because the primary outcome was defined a priori, no adjustment for multiple testing was conducted [10]. The secondary analyses were explorative; therefore, the results for secondary outcomes are judged in an exploratory manner. Sensitivity analyses on the variances of diagnosis-specific cost-effectiveness differences were operationalized using nonparametric bootstrapping [28]. Based on these bootstrap results, cost-effectiveness acceptance curves were calculated to show probabilities of cost-effectiveness according to different values of the willingness to pay for a QALY gained. Since no German cost-effectiveness threshold exist, we oriented on international thresholds and defined an hypothetical ICER below 50,000€ as being cost-effective [29]. Due to the uncertainty of this threshold and the general discussion regarding cost-effectiveness thresholds, the above mentioned cost-effectiveness probabilities were calculated [30, 31]. The data analyses were independently performed with SAS for Windows version 9.2 or higher (SAS Institute, Cary, NC, USA) and SPSS for Windows or Mac version 23 (SPSS Inc., Chicago, IL, USA) by two researchers.

## Results

### Baseline characteristics

**Fig. 1:**
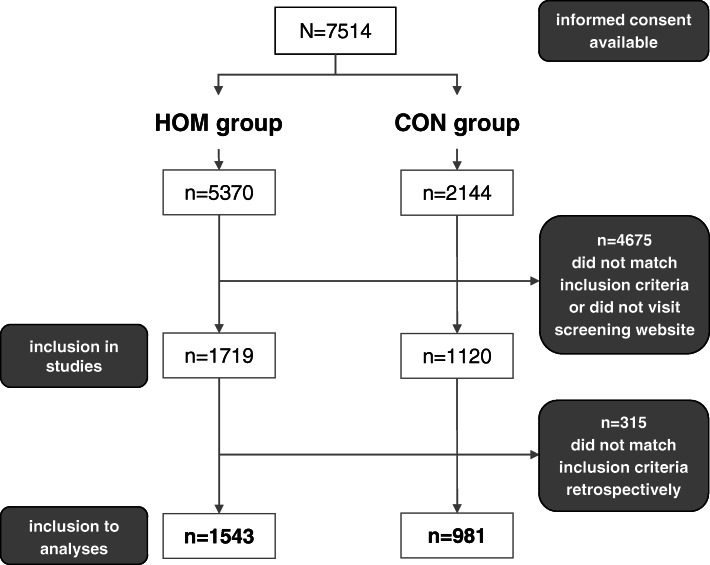
Flowchart of participant inclusion

A total of 7514 individuals provided informed consent (5370 in the ICCH), 4675 had to be rejected (617 potential participants did not participate in the screening, the remaining 4058 did not meet the eligibility criteria). Another 315 individuals were excluded during data analyses due to a mismatch between self-reported and documented health data diagnoses. Between the baseline questionnaires and 12 months of follow-up 8.5% in the HOM and 3.8% in the CON group were lost to follow-up. A total of 2524 participants (1543 HOM group, 981 CON group) were included in the present analyses (see Fig. 1).

The matching resulted in comparable age and sex distributions in both groups (see Table 2). However, the HOM group was higher educated and utilized complementary medicine more often in the 12 months prior to baseline. Additionally, the distribution among study diagnoses as well as states of residence, various baseline effectiveness measures and diagnosis-specific costs prior to baseline were different among the groups. The proportion of participants in the HOM group who received their initial consultation as intended by the study protocol was only 32.1%. In both groups, the majority of participants were diagnosed with depression (29.3% of the HOM group and 34.8% of the CON group). Diagnosis-specific effectiveness measures indicate a higher disease severity over all study diagnoses at baseline in the HOM group, with the exception of participants diagnosed with asthma. The mean total costs in the 12 months prior to baseline were higher in the CON group for participants diagnosed with migraine or headache, allergic rhinitis (both perspectives) and depression (insurer perspective). Higher total costs prior to baseline in the HOM group were observed for participants diagnosed with asthma, atopic dermatitis (both perspectives) and depression (societal perspective). Among the study diagnoses, depression was associated with the highest total costs prior to baseline in both groups and from both perspectives.

**Table 2:** Baseline characteristics of study participants. Data include the mean (±SD) or the number of persons (%)

	HOM group (***n*** = 1543)	CON group (***n*** = 981)
Age (years)	43.1 (±13.6)	43.0 (±12.9)
Women	1208 (78.3)	773 (78.8)
Education, A level or similar	965 (62.5)	542 (55.2)
Utilization of complementary medicine 12 months prior to baseline	955 (61.9)	119 (12.1)
Per-protocol initial anamnesis in HOM group	496 (32.1)	N/A
State of residence (n (%))		
Abroad	2 (0.1)	0 (0)
Baden-Wuerttemberg	185 (12)	105 (10.7)
Bavaria	206 (13.4)	109 (11.1)
Berlin	188 (12.2)	59 (6.0)
Brandenburg	40 (2.6)	33 (3.4)
Bremen	14 (0.9)	10 (1.0)
Hamburg	103 (6.7)	56 (5.7)
Hesse	118 (7.6)	106 (10.8)
Mecklenburg-Western Pomerania	24 (1.6)	14 (1.4)
Lower Saxony	123 (8.0)	102 (10.4)
North-Rhine Westphalia	300 (19.4)	224 (22.8)
Rhineland-Palatinate	87 (5.6)	49 (5.0)
Saarland	7 (0.5)	7 (0.7)
Saxony	22 (1.4)	32 (3.3)
Saxony-Anhalt	18 (1.2)	13 (1.3)
Schleswig-Holstein	90 (5.8)	49 (5.0)
Thuringia	16 (1.0)	13 (1.3)
Diagnosis and specific measures		
*Migraine/headache (G43.9/ R51)*	422 (27.3)	266 (27.1)
Days with headache (last 4 weeks)	7.2 (±6.1)	6.2 (±5.6)
Utility value^a^	0.683 (±0.118)	0.711 (±0.125)
Total costs previous 12 months in € (insurer perspective)	1080.82 (±1064.27)	1138.07 (±938.70)
Total costs previous 12 months in € (societal perspective)	4399.99 (±8472.08)	4476.86 (±7713.43)
*Allergic rhinitis (J30.1)*	317 (20.5)	188 (19.2)
RQLQ(S) score	2.5 (±1.3)	1.8 (±1.3)
AdolRQLQ score	2.0 (±1.0)	2.1 (±1.1)
Utility value^a^	0.727 (±0.124)	0.788 (±0.118)
Total costs previous 12 months in € (insurer perspective)	912.31 (±872.55)	973.18 (±726.53)
Total costs previous 12 months in € (societal perspective)	2716.87 (±5866.87)	2867.22 (±5675.07)
*Asthma (J45.9)*	123 (8.0)	81 (8.3)
AQLQ(S) score	5.1 (±0.9)	5.5 (±1.1)
PAQLQ score	4.3 (±1.1)	5.8 (±0.8)
Utility value^a^	0.750 (±0.123)	0.788 (±0.126)
Total costs previous 12 months in € (insurer perspective)	1233.89 (±1226.28)	1182.97 (±1071.87)
Total costs previous 12 months in € (societal perspective)	3913.54 (±7459.30)	3191.32 (±5544.62)
*Atopic dermatitis (L20)*	229 (14.8)	105 (10.7)
DLQI score	8.9 (±6.1)	6.1 (±5.4)
CDLQI score	6.6 (±4.3)	2.8 (±2.9)
Utility value^a^	0.743 (±0.128)	0.788 (±0.119)
Total costs previous 12 months in € (insurer perspective)	929.83 (±874.23)	834.08 (±805.37)
Total costs previous 12 months in € (societal perspective)	3150.44 (±7876.47)	2644.33 (±3251.03)
*Depression (F32.9)*	452 (29.3)	341 (34.8)
BDI-II Score	23.3 (±10.0)	20.3 (±11.3)
Utility value^a^	0.621 (±0.103)	0.666 (±0.123)
Total costs previous 12 months in € (insurer perspective)	1666.76 (±1821.34)	1737.11 (±1091.65)
Total costs previous 12 months in € (societal perspective)	10,679.85 (±17,270.82)	7634.41 (±11,252.44)

^a^Derived from the SF-12

### Effectiveness

The results of the primary effectiveness analyses are displayed in Table 3. Statistically significant mean group differences for the primary outcome after 6 months were observed for participants with migraine or headache (Δ-days with headache: − 0.9, *p* = 0.042; HOM group perspective), asthma (Δ-AQLQ(S): + 0.4 points, *p* = 0.014; HOM group perspective) and depression (Δ-BDI-II: − 5.6 points, *p* ≤ 0.001; HOM group perspective), all in favor of the HOM group. Nevertheless, the statistically significant differences must be interpreted with reference to the clinical relevance of the effect. Minimal clinical important difference (MID) was only found for depression (MID for the BDI-II: 5.0 points) [21], but not for migraine or headache (according to common expert opinion) and asthma (MID for the AQLQ(S): 0.5 points). Due to the small number of adolescent participants, no specific analyses for adolescent participants diagnosed with allergic rhinitis, asthma and atopic dermatitis were performed.

**Table 3:** Adjusted means (95% CI) of diagnosis-specific primary effectiveness measures, diagnosis-specific group differences and minimal clinical important differences, 6 months of follow-up

	HOM group		CON group				
Diagnosis	N	Adj. mean (95% CI)	N	Adj. mean (95% CI)	Group Δ*	*p*-value^†^	Minimal clinical important difference^‡^ reached?
Migraine/headache *Days with headache (last 4 weeks)*	400	3.7 (2.2–5.2)	266	4.6 (2.9–6.3)	−0.9	0.042	No
Allergic rhinitis *RQLQ(S) score*	283	1.6 (1–2.2)	167	1.8 (1.2–2.5)	−0.2	0.209	No
Asthma *AQLQ(S) score*	111	5.8 (5.4–6.3)	66	5.4 (4.9–5.9)	0.4	0.014	No
Atopic dermatitis *DLQI score*	206	6.8 (4.5–9.1)	97	8.2 (5.5–10.9)	− 1.4	0.086	No
Depression *BDI-II score*	423	14.1 (11.9–16.3)	334	19.7 (17.2–22.2)	−5,6	< 0.001	Yes

*HOM group minus CON group, ^†^ adjusted for age, sex, education, residential state, complementary medicine affiliation, per protocol initial anamnesis, baseline value of diagnosis specific scores, ^‡^Minimal clinical important differences refer to the diagnoses specific primary effectiveness outcomes (see Table 1), judgement for migraine/headache depends on expert opinion

**Fig. 2:**
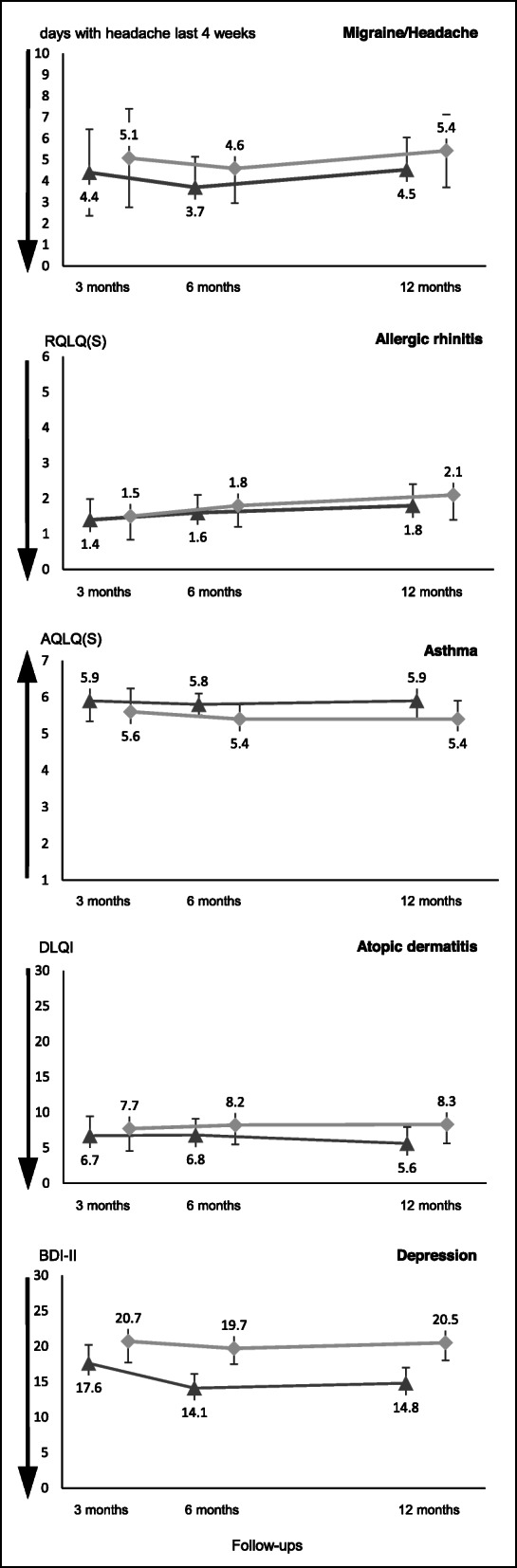
Adjusted diagnosis-specific effectiveness measures progression by group. Legend: Error bars denote 95% CIs. Effectiveness measures are adjusted for specific baseline value, age, sex, formal education, state of residence, use of complementary medicine in the 12 months prior to baseline and protocol-compliant initial anamnesis. Triangles represent the HOM group, and dots represent the CON group. Arrows reflect the direction of effect improvements on the diagnosis-specific scales

Figure 2 also shows the adjusted diagnosis-specific effectiveness scores after baseline and contains the secondary effectiveness outcomes of both groups after 3 and 12 months.

### Costs and cost-effectiveness

Over the 12-month period, adjusted total costs were statistically significant higher in the HOM group from the insurer perspective for participants diagnosed with migraine or headache (Δ total costs per insured: +423.13€; *p* < 0.001), asthma (Δ total costs: +740.45€; *p* < 0,001) and depression (Δ total costs: +420.67€; *p* < 0.001) (see Table 4). Adjusted total cost in the HOM group for participants diagnosed with allergic rhinitis (Δ total costs: + 99.22€; *p* = 0.220) and atopic dermatitis (Δ total costs: + 139.49€; *p* = 0.250) were also higher but not found to be statistically significant. From the societal perspective, the directions of adjusted mean total cost group differences varied among diagnoses.

**Table 4:** Diagnosis-specific adjusted means of total costs over 12 months after baseline, insurer and secondary societal perspectives

	HOM group		CON group			
Diagnosis	N Cost utilization	Adj. mean (€) (95% CI)	N Cost utilization	Adj. mean (€) (95% CI)	Group Δ*	*p*-value†
**Insurer perspective (primary cost outcome)**						
Migraine/headache	422	1654.42 (1309.80–1999.04)	265	1231.30 (839.57–1623.02)	423.13	< 0.001
Allergic rhinitis	316	1137.50 (880.82–1394.17)	187	1038.28 (748.78–1327.79)	99.22	0.220
Asthma	123	1463.28 (938.27–1988.29)	80	722.83 (116.94–1328.73)	740.45	< 0.001
Atopic dermatitis	229	1269.32 (962.23–1576.40)	104	1129.83 (763.50–1496.17)	139.49	0.250
Depression	452	1974.63 (1703.83–2245.43)	341	1553.96 (1241.84–1866.08)	420.67	< 0.001
**Societal perspective**						
Migraine/headache	422	4938.64 (2358.01–7519.28)	265	5010.48 (2077.35–7943.61)	−71,84	0.924
Allergic rhinitis	316	3073.44 (1125.04–5021.84)	187	3274.15 (1074.55–5473.74)	−200,71	0.744
Asthma	123	3022.62 (−43.81–6089.04)	80	1285.69 (−2244.58–4815.95)	1736,93	0.157
Atopic dermatitis	229	7353.94 (5070.56–9637.31)	104	7536.04 (4812.85–10,259.24)	−182,1	0.839
Depression	452	9402.39 (6182.19–12,622.59)	341	7207.43 (3499.95–10,914.90)	2194,96	0.078

* HOM group minus CON group, † adjusted for age, sex, education, residential state, complementary medicine affiliation, per protocol initial anamnesis, baseline utility value, total costs 12 months prior to baseline

Adjusted mean group QALY differences were statistically significant in favor of the HOM group for the following diagnoses: migraine or headache, atopic dermatitis and depression. No statistically significant differences were obtained for allergic rhinitis and asthma (see Table 5).

**Table 5:** Adjusted means of QALYs (95% CI) over 12 months after baseline

	HOM group		CON group			
Diagnosis	N	Adj. mean (95% CI)		Adj. mean (95% CI)	Group Δ*	*p*-value^†^
Migraine/headache	422	0.727 (0.700–0.754)	262	0.693 (0.662–0.724)	0.034	< 0.001
Allergic rhinitis	317	0.782 (0.754–0.811)	188	0.773 (0.741–0.805)	0.009	0.291
Asthma	123	0.762 (0.726–0.798)	81	0.774 (0.733–0.816)	−0.012	0.379
Atopic dermatitis	229	0.761 (0.734–0.787)	105	0.723 (0.692–0.755)	0.038	< 0.001
Depression	452	0.673 (0.654–0.692)	341	0.638 (0.616–0.660)	0.035	< 0.001

*HOM group minus CON group, ^†^ adjusted for age, sex, education, residential state, complementary medicine affiliation, per protocol initial anamnesis, baseline utility value

**Fig. 3:**
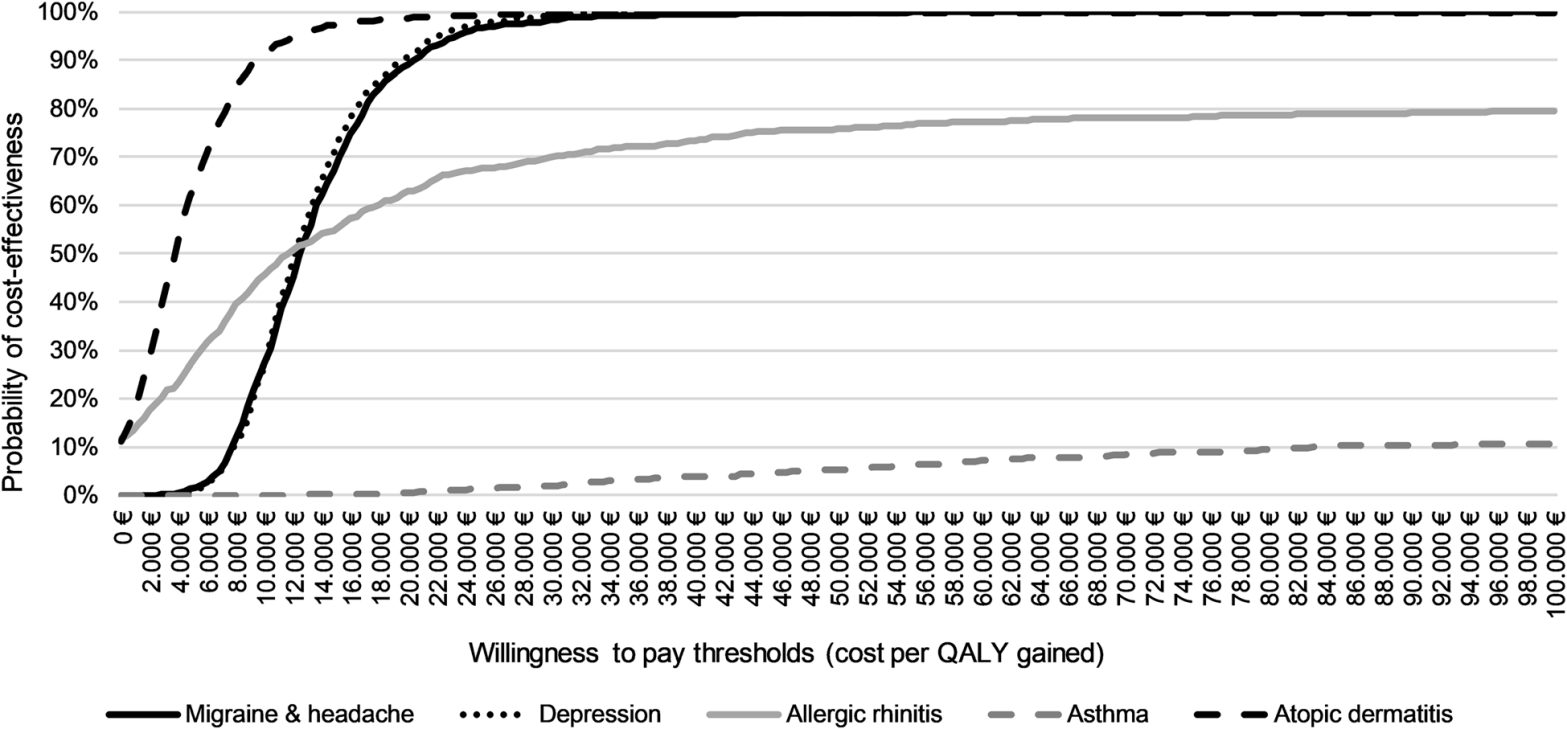
Cost-effectiveness acceptance curves of treatment with an additional enrollment to ICCH versus usual care alone on diagnosis-specific bootstrapping (1000 estimates). Legend: ICCH, integrated care contract homeopathy; QALYs, quality-adjusted life years

Because of more QALYs and higher costs in the HOM group, ICERs were calculated from the insurer perspective for participants diagnosed with migraine or headache (ICER: 12,309€ [95% CI 11,953-12,665] per QALY gained) and depression (ICER: 11,879€ [95% CI 11,580-12,178] per QALY gained). Furthermore, the statistically significant QALY differences in favor of the HOM group indicate cost-effectiveness from the insurer perspective for the treatment of participants with atopic dermatitis (ICER: 3761 [95% CI 3435-4087] per QALY gained). In addition, cost-effectiveness acceptance curves from the insurer perspective are shown in Fig. 3, based on diagnosis-specific bootstrapping with 1000 estimates.

From the societal perspective, the cost-effectiveness of an additional enrollment to the ICCH is indicated for participants with migraine or headache and atopic dermatitis due to statistically significant QALY differences and statistically non-significant cost savings (all in favor of the HOM group). For participants with depression, statistically non-significant higher costs were associated with QALY gains for the HOM group. Nevertheless, an ICER of 59,744€ [95% CI 57,245-62,243] per QALY gained did not support cost-effectiveness from the societal perspective.

## Discussion

### Key results

The primary diagnosis-specific effectiveness analyses showed an additional effect in the HOM group for participants diagnosed with migraine or headache, asthma and depression, with the effect for depression being minimally clinically relevant after 6 months.

From the primary economic perspective (insurer), the HOM group participants with migraine or headache, asthma and depression had statistically significant higher mean total costs compared to the CON group over the 12-month period. After relating costs from the insurance perspective with QALYs, treatment after an additional enrollment in the ICCH seems to be cost-effective for participants with migraine or headache, atopic dermatitis and depression but not for those with allergic rhinitis and asthma.

### Strength and limitations

The study reflected a routine care situation and applied well-known diagnosis-specific and cross-diagnosis questionnaires to assess the effectiveness and utility values for QALY measurement. The health claims data used for cost analysis were derived from one of the largest nationwide operating statutory health insurances in Germany.

The most relevant limitations of the presented analyses resulted from the observational study design. Without randomization, no random (and thus by chance) distribution of baseline characteristics was possible. Additionally, the recruitment of the CON group participants was based on comparatively few matching parameters. For practical and procedural reasons of study conduct, propensity score matching or entropy balancing were not feasible. Effectiveness measures and total costs were adjusted for a number of known and identified diagnosis-specific socioeconomic variables, effectiveness measures at baseline and total cost prior to baseline. Thereby, the total cost prior to baseline were used as a proxy, e.g. for comorbidities and hospitalization. However, as a result of the partly aggregated available cost dataset, we were not able to assess if and to what extend psychotherapy or other effective forms of outpatient treatment was used in both groups. And there might be other group differences (e.g., symptom-durations, health behaviors, urbanization of the region of residence, expectations and physician-patient interactions) that are completely unknown and could therefore not be taken into account in the analyses. Thus, it is not unlikely that the findings are driven by unknown confounding factors [32]. Likewise, participants in the HOM group had worse diagnosis specific baseline scores and utility values over all study diagnoses in comparison to the CON group indicating a higher disease related suffering when visiting the homeopathic physician at the time of study inclusion. Although we applied ANCOVAs and adjusted for baseline values a regression to the mean effect is not unlikely [33]. Moreover, it was beyond the scope of the study to address the utilization of specific ICCH components or the unknown individual globule intake. Hence, from this study, it is not possible to draw any conclusion about specific ICCH components.

Furthermore, in approximately two-thirds of the participants in the HOM group (67.1%), the study protocol was violated, and participants received their initial homeopathic consultation before completing the baseline questionnaire; therefore, we had to include the per protocol initial anamnesis in the statistical model as a further binary covariate.

Further limitations included a much smaller sample than planned and an unbalanced number of participants in the HOM vs. the CON groups. Initially, significantly more matched controls were planned. This problem could not be resolved even by inviting up to 20 potential matched controls. The low response rate, especially in the CON group, might be reflected upon the missing of incentives for study participation. In combination with the different recruitment approaches in both groups, recruitment bias cannot be ruled out. Yet, the majority of observable characteristics were comparable at baseline and as describes above, we adjusted for a number of relevant and available variables. The smaller numbers of adolescent participants did not allow separate analyses for this subgroup. We decided to combine the participants with migraine and headaches into one group because of overlapping self-reported diagnoses and small sample sizes. In addition, no MID for days with headache has been defined. Therefore, we had to rely on the expert opinion of an experienced neurologist when judging clinical relevance.

As already described by Ostermann et al., the usage of cost data from only one health insurance company limits the generalizability of the findings [9]. The secondary cost-effectiveness analyses from a societal perspective can be assumed to systematically underestimate the total costs. Although the gross costs provided by insurance company were considered and a monetary valuation of the number of days of being unable to work was performed, it is likely that the chronically ill participants had unobserved expenses for non-refundable over-the-counter products and treatments [34, 35].

### Interpretation

While taking the effectiveness and cost-effectiveness of treatment after an additional enrollment to the ICCH into account, the present study added some information to our previous cost comparison analyses [8, 9]. These previous studies used a different and larger sample but were retrospective and did not include outcome data. The cost results of this prospective study confirmed the additional costs from the insurer perspective; however, in contrast to the previous analyses no statistically significant cost differences between groups from a societal perspective were found. One explanation could be that the previous studies had a much larger sample and used propensity score matching, which allowed adjustments for many more characteristics than in our present study and was therefore more likely to reduce confounding.

Our results confirm the inconsistent results in the published literature. For example, the systematic review by Viksveen et al. regarding the cost-effectiveness of homeopathic treatment identified 14 studies. While eight studies reported improved effects combined with cost savings, four studies showed similar or improved outcomes at the same costs, and two studies showed similar effects combined with higher costs from the homeopathic perspective [10]. However, most studies were small and had methodological limitations. Additionally, previous systematic literature reviews examining the effectiveness or cost-effectiveness of homeopathic treatment for different chronic diseases faced many studies of rather low scientific quality and were therefore unable to draw overall conclusions [10, 36–39]. The authors of one review facing these problems while assessing the effects of homeopathy for patients with asthma demanded observational studies to reveal the effects of homeopathic treatment beyond the intervention [37]. With our observational study in a routine care setting, we addressed this issue and were able to investigate not only the effects but also the cost-effectiveness of treatment after an additional enrollment in an ICCH. Nevertheless, because of the limitations discussed above and the variance of the results over the included diagnoses, our study is not able to provide a clearer answer for the general cost-effectiveness of an additional homeopathic treatment.

A main component of the examined ICCH is the detailed and extensive homeopathic case history. According to 2002 data, a usual general practitioner in Germany spends an average of only 7.6 min on each patient consultation [40]; this finding allows us to hypothesize that larger improvements in the HOM group could also be attributed to an improved physician-patient relationship and better overall care. However, the time a physician spends with a patient might have more influence on the patient’s general quality of life (which is reflected by our QALY assessment) than on diagnosis-specific complaints, which might explain that we conclude cost-effectiveness in some subgroups without detecting clinically relevant differences in diagnosis-specific endpoints.

## Conclusion

After an additional enrollment in the ICCH, the treatment of participants with depression showed clinically relevant improvements, but not the treatment of participants with migraine or headache, asthma, allergic rhinitis and atopic dermatitis. From an insurer perspective, treatment with additional ICCH enrollment resulted in higher costs over all study diagnoses but seemed to be cost-effective in terms of additional cost per QALY gained for migraine and headache, atopic dermatitis and depression. Because of the nature of the observational non-randomized study design and further limitations, our findings should be considered cautiously and no conclusions regarding the effectiveness of specific treatment components can be made. There are evidence based treatments available for the evaluated diagnoses, therefore, the results of this study should be seen with caution and critically reflected within the respective health care context. Further research is needed to overcome limitations of this study and to confirm our findings.

## Data Availability

The data that support the findings of this study are available from the German statutory health insurance company Techniker Krankenkasse but restrictions apply to the availability of these data, which were used under license for the current study, and so are not publicly available. Data are however available from the authors upon reasonable request and with permission of the German Federal (Social) Insurance Office and the Techniker Krankenkasse.

## Abbreviations

ICCH: Integrated care contract for homeopathy
HOM: Participant in the intervention group
CON: Matched control participant
QALY: Quality-adjusted life year
RQLQ(S): Standardised rhinoconjunctivitis quality of life questionnaire
AdolRQLQ: Adolescent rhinoconjunctivitis quality of life questionnaire
AQLQ(S): Standardised asthma quality of life questionnaire
PAQLQ: Paediatric asthma quality of life questionnaire
DLQI: Dermatology life quality index
CDLQI: Children dermatology life quality index
BDI-II: Beck depression inventory II
SF-12: 12-Item short form health survey version 1
ICER: Incremental cost-effectiveness ratio
SD: Standard deviation
CI: Confidence interval
ANCOVA: Covariance analysis
MID: Minimal clinical important difference

## References

[1] Maddox J, Randi J, Stewart WW (1988). "high-dilution" experiments a delusion. Nature..

[2] Smith K (2012). Against homeopathy--a utilitarian perspective. Bioethics..

[3] Jonas WB, Kaptchuk TJ, Linde K (2003). A critical overview of homeopathy. Ann Intern Med.

[4] Ullman D. The homeopathic revolution: why famous people and cultural heroes choose homeopathy: North Atlantic books; 2007.

[5] De Sombre S. Homöopathische Arzneimittel 2014. Allensbach: Institut für Demoskopie Allensbach; 2014.

[6] Relton C, Cooper K, Viksveen P, Fibert P, Thomas K (2017). Prevalence of homeopathy use by the general population worldwide: a systematic review. Homeopathy.

[7] Managementgesellschaft des Deutschen Zentralvereins hömöopathischer Ärzte mbH. Teilnehmende Gesetzliche Krankenkassen: Managementgesellschaft des DZVhÄ mbH; 2018. Available from: https://www.managementgesellschaft-dzvhae.de/selektivvertraege-homoeopathie-vertragsteilnehmer/teilnehmende-gesetzliche-krankenkassen/.

[8] Ostermann JK, Reinhold T, Witt CM (2015). Can additional homeopathic treatment save costs? A retrospective cost-analysis based on 44500 insured persons. PLoS One.

[9] Ostermann JK, Witt CM, Reinhold T (2017). A retrospective cost-analysis of additional homeopathic treatment in Germany: long-term economic outcomes. PLoS One.

[10] Viksveen P, Dymitr Z, Simoens S (2014). Economic evaluations of homeopathy: a review. Eur J Health Econ.

[11] Witt CM, Brinkhaus B, Pach D, Reinhold T, Wruck K, Roll S (2009). Homoeopathic versus conventional therapy for atopic eczema in children: medical and economic results. Dermatology.

[12] Roll S, Reinhold T, Pach D, Brinkhaus B, Icke K, Staab D (2013). Comparative effectiveness of homoeopathic vs. conventional therapy in usual care of atopic eczema in children: long-term medical and economic outcomes. PloS One.

[13] Jena S, Witt C, Brinkhaus B, Wegscheider K, Willich S (2008). Acupuncture in patients with headache. Cephalalgia.

[14] Juniper EF, Thompson AK, Ferrie PJ, Roberts JN (1999). Validation of the standardized version of the Rhinoconjunctivitis quality of life questionnaire. J Allergy Clin Immunol.

[15] Juniper EF, Buist AS, Cox FM, Ferrie PJ, King DR (1999). Validation of a standardized version of the asthma quality of life questionnaire. Chest.

[16] Juniper EF, Guyatt GH, Feeny DH, Ferrie PJ, Griffith LE, Townsend M (1996). Measuring quality of life in children with asthma. Qual Life Res.

[17] Juniper EF, Howland WC, Roberts NB, Thompson AK, King DR (1998). Measuring quality of life in children with rhinoconjunctivitis. J Allergy Clin Immunol..

[18] Finlay AY, Khan GK (1994). Dermatology life quality index (DLQI)--a simple practical measure for routine clinical use. Clin Exp Dermatol.

[19] Lewis-Jones MS, Finlay AY (1995). The Children's dermatology life quality index (CDLQI): initial validation and practical use. Br J Dermatol.

[20] Beck AT, Ward CH, Mendelson M, Mock J, Erbaugh J (1961). An inventory for measuring depression. Arch Gen Psychiatry.

[21] Button KS, Kounali D, Thomas L, Wiles NJ, Peters TJ, Welton NJ (2015). Minimal clinically important difference on the Beck depression inventory--II according to the patient's perspective. Psychol Med.

[22] Bullinger M, Kirchberger I (1998). SF-36 Fragebogen zum Gesundheitszustand.

[23] Sanders GD, Neumann PJ, Basu A, Brock DW, Feeny D, Krahn M (2016). Recommendations for conduct, methodological practices, and reporting of cost-effectiveness analyses: second panel on cost-effectiveness in health and medicine. Jama..

[24] Brazier JE, Roberts J (2004). The estimation of a preference-based measure of health from the SF-12. Med Care.

[25] Richardson G, Manca A (2004). Calculation of quality adjusted life years in the published literature: a review of methodology and transparency. Health Econ.

[26] Hanly P, Timmons A, Walsh PM, Sharp L (2012). Breast and prostate cancer productivity costs: a comparison of the human capital approach and the friction cost approach. Value Health.

[27] Statistisches Bundesamt. Jahresschätung Arbeitskosten [Internet]. Wiesbaden: Statistisches Bundesamt; 2019. Available from: https://www-genesis.destatis.de/genesis/online/link/tabelleErgebnis/62431-0001.

[28] Efron B (1979). Bootstrap methods: another look at the jackknife. Ann Stat.

[29] Simoens S (2010). How to assess the value of medicines?. Front Pharmacol.

[30] Neumann PJ, Cohen JT, Weinstein MC (2014). Updating cost-effectiveness — the curious resilience of the $50,000-per-QALY threshold. N Engl J Med.

[31] Cameron D, Ubels J, Norström F (2018). On what basis are medical cost-effectiveness thresholds set? Clashing opinions and an absence of data: a systematic review. Glob Health Action.

[32] Skelly AC, Dettori JR, Brodt ED (2012). Assessing bias: the importance of considering confounding. Evid Based Spine-Care J.

[33] Barnett AG, van der Pols JC, Dobson AJ (2005). Regression to the mean: what it is and how to deal with it. Int J Epidemiol.

[34] Bridevaux IP (2004). A survey of patients’ out-of-pocket payments for complementary and alternative medicine therapies. Complement Ther Med.

[35] Barrenberg E, Knopf H, Garbe E. Over-The-Counter (OTC) Drug Consumption among Adults Living in Germany: Results from the German Health Interview and Examination Survey for Adults 2008-2011 (DEGS1). Pharmacy (Basel). 2018;6(2):52. 10.3390/pharmacy6020052. 10.3390/pharmacy6020052 PMC6024976 29880765

[36] Owen JM, Green BN. Homeopathic treatment of headaches: a systematic review of the literature. J Chiropr Med. 2004;3(2):45–52. 10.1016/S0899-3467(07)60085-8 PMC2646987 19674623

[37] McCarney RW, Linde K, Lasserson TJ. Homeopathy for chronic asthma. Cochrane Database Syst Rev. 2004;1. 10.1002/14651858.CD000353.pub2 PMC7032670 14973954

[38] Banerjee K, Mathie RT, Costelloe C, Howick J (2017). Homeopathy for allergic rhinitis: a systematic review. J Altern Complement Med.

[39] Viksveen P, Fibert P, Relton C (2018). Homeopathy in the treatment of depression: a systematic review. Eur J Integr Med.

[40] Deveugele M, Derese A, van den Brink-Muinen A, Bensing J, De Maeseneer J (2002). Consultation length in general practice: cross sectional study in six European countries. BMJ.

