# Correction to: Effectiveness and cost-effectiveness of treatment with additional enrollment to a homeopathic integrated care contract in Germany

**DOI:** 10.1186/s12913-020-05766-6

**Published:** 2020-09-30

**Authors:** Benjamin Kass, Katja Icke, Claudia M. Witt, Thomas Reinhold

**Affiliations:** Institute for Social Medicine, Epidemiology and Health Economics, Charité - Universitätsmedizin Berlin, corporate member of Freie Universität Berlin, Humboldt-Universität zu Berlin, and Berlin Institute of Health, Berlin, Germany

**Correction to: BMC Health Serv Res 20, 872 (2020)**

**https://doi.org/10.1186/s12913-020-05706-4**

Following publication of the original article [1], the authors would like to remove the incorrect group difference for atopic dermatitis in the Abstract. The correct group difference of Δ-DLQI: − 1.4, *p* = 0.086 is neither clinically relevant nor statistically significant. The correct value is stated in the main text and discussed in the correct way.

The sentence currently reads:

The primary effectiveness outcomes after six months were statistically significant in favor of the HOM group for migraine or headache (Δ = difference between groups, days with headache: − 0.9, *p* = 0.042), asthma (Δ-AQLQ(S): + 0.4, *p* = 0.014), atopic dermatitis (Δ- DLQI: − 5.6, *p* ≤ 0.001) and depression (Δ-BDI-II: − 5.6, *p* ≤ 0.001).

The sentence should read:

The primary effectiveness outcomes after six months were statistically significant in favor of the HOM group for migraine or headache (Δ = difference between groups, days with headache: − 0.9, *p* = 0.042), asthma (Δ-AQLQ(S): + 0.4, *p* = 0.014) and depression (Δ-BDI-II: − 5.6, *p* ≤ 0.001).
